# *In-Vivo* Retention of 5-Fluorouracil Using ^19^F Magnetic Resonance Chemical Shift Imaging in Colorectal Cancer in a Murine Model

**DOI:** 10.1038/s41598-019-49716-7

**Published:** 2019-09-13

**Authors:** Yurii Shepelytskyi, Matthew S. Fox, Karen Davenport, Tao Li, Mitchell S. Albert, Eric Davenport

**Affiliations:** 1 Chemistry and Materials Science program, Lakehead University, 955 Oliver Rd., Thunder Bay, ON P7B 5E1 Canada; 2 Lawson Health Research Institute, 750 Base Line Road East, London, ON N6C 2R5 Canada; 3 Thunder Bay Regional Health Research Institute, 980 Oliver Rd., Thunder Bay, ON P7B 5E1 Canada; 4 Chemistry Department, Lakehead University, 955 Oliver Rd., Thunder Bay, ON P7B 5E1 Canada; 5 Northern Ontario School of Medicine, 955 Oliver Rd., Thunder Bay, ON P7B 5E1 Canada; 6 Thunder Bay Regional Health Science Centre, 980 Oliver Rd., Thunder Bay, ON P7B 6V4 Canada

**Keywords:** Magnetic resonance imaging, Cancer therapeutic resistance, Colon cancer

## Abstract

Colorectal cancer is the third leading cause of cancer death worldwide. 5-Fluorouracil (5-FU) is one of the most commonly used chemotherapies for treatment of solid tumours, including colorectal cancer. The efficacy of treatment is dependent on tumour type and can only be determined six weeks after beginning chemotherapy, with only 40–50% of patients responding positively to the 5-FU therapy. In this paper, we demonstrate the potential of using Magnetic Resonance (MR) Chemical Shift Imaging (CSI) for *in-vivo* monitoring of 5-FU tumor-retention in two different colorectal tumour types (HT-29 & H-508). Time curves for 5-FU signals from the liver and bladder were also acquired. We observed significant differences (*p* < 0.01) in 5-FU signal time dependencies for the HT-29 and H-508 tumours. Retention of 5-FU occurred in the H-508 tumour, whereas the HT-29 tumour is not expected to retain 5FU due to the observation of the negative b time constant indicating a decline in 5FU within the tumour. This study successfully demonstrates that CSI may be a useful tool for early identification of 5-FU responsive tumours based on observed tumour retention of the 5-FU.

## Introduction

Colorectal cancer is the third most common cause of cancer death worldwide according to the World Healthcare Organization^1^ and is the second most commonly diagnosed cancer in Canada according to the Canadian Cancer Society^2^. Colorectal cancer is responsible for approximately 14% of new cancer cases and 12% of cancer deaths in Canada^3^. According to Canadian Cancer Statistics (2016), 7% of men and 6% of women are expected to develop colorectal cancer during their lifetimes^2^. Moreover, an increasing incidence of colorectal cancer among young adults in Canada has been observed^4^.

5-Fluorouracil (5-FU) is one of the most widely used cytotoxic chemotherapies for treatment of a variety of solid tumours, including colorectal and breast cancer^5–9^. However, the treatment response rate (percentage of patients whose tumour shrinks or disappeared after treatment) for 5-FU based chemotherapy are relatively low^5,10^. Biochemical modulation of 5-FU has increased response rates up to 40–50%^5,11,12^, while the influence on overall survival has been limited^8^. The improvement in overall survival was observed when 5-FU was modulated with irinotecan^13,14^. The current colorectal cancer mortality rates reflect – at least in part – resistance of individual tumours to 5-FU treatment. It has been clinically demonstrated that the “trapping phenomenon” (the half-life of drug in the tumour is longer than 20 minutes) correlates with the clinical effectiveness of 5-FU chemotherapy^15^. Thus, a method to detect the responsiveness of a tumor to 5-FU in the earliest stages of treatment (i.e., sooner than six weeks) may enable effective targeting of 5-FU therapy to responsive tumours and also may prevent unnecessary exposure to cytotoxic chemotherapy in patients with 5-FU resistant tumours.

It is hoped that early identification of 5-FU resistant colorectal tumours will enable oncologists to choose treatment strategies more likely to improve patient survival and minimize unnecessary morbidity.

Several previous studies using MRI were conducted using Diffusion-Weighted Imaging (DWI) for early detection of the treatment response in patients with colorectal cancer^16–18^. It was found that the increase in Apparent Diffusion Coefficient (ADC) of tissue water within the tumour was significantly higher in responders compared to non-responders^16^. Marugami *et al*. was able to distinguish responders from non-responders 9 days after the initiation of chemotherapy based on the ADC changes in liver methastasis^17^. However, patients already received two infusions of 5-FU. Similar result was obtained by Lavdas *et al*.^18^. Although these results can be considered as an early detection of the tumour response, there are several limitations associated with DWI. Due to power requirements, hardware limitations and other external factors DWI accuracy is limited and the image quality is low^19^. Furthermore, DWI image are often susceptible to various artifacts like ghosting, blurring, ringing, distortions etc^19,20^.

It is hypothesized that the early monitoring of 5-FU retention at the site of colorectal tumours can indicate the tumour responsiveness to 5-FU chemotherapy. 5-FU metabolizes into fluorinated nucleotides (Fnuc) and α-fluoro-β-alanine (Fbal) that each display a different chemical shift (i.e., 5-FU and its metabolites have different resonant frequencies in the ^19^F magnetic resonance (MR) spectrum^21–28^. All of them can be visualized using Magnetic Resonance Imaging (MRI). Chemical-Shift Selective images of 5-FU and its metabolites have been previously acquired in a rat model^22–25^ and in a mouse model^26–28^. The high natural abundance (approximately 100%) and large gyromagnetic ratio of Fluorine-19 (^19^F) lead to a strong observed signal of 5-FU. ^19^F MRI is a non-invasive and non-ionizing imaging technique. Another significant benefit of using ^19^F MRI is the absence of fluorinated compounds in the human body, thus there is no natural background signal. These characteristics together, make ^19^F MRI a promising method for monitoring 5-FU retention immediately following a single chemotherapy treatment. This would be a significant improvement to the current method for evaluating the efficacy of chemotherapy based on the observation of reduced tumour size after prescribed chemotherapy treatment.

Chemical Shift Imaging (CSI) is an extension of MR spectroscopy (MRS), allowing metabolite information to be measured. This technique has already been used for imaging the small intestines of mice with orally administrated 5-FU^28^. Also, CSI has been used for imaging the liver of patients with colorectal cancer and breast cancer^29^ and to study the metabolism of 5-FU in liver metastasis^30,31^. Although the metabolism of 5-FU was studied within tumours^27,28,32^ and liver^29^, the CSI imaging technique of 5-FU was not used for tumour resistivity detection. In addition, ^19^F CSI of 5-FU was not implemented as a clinical diagnostic modality.

The purpose of this study was to determine if there were observable differences between the signal to noise ratio (SNR) from colorectal tumours that are insensitive (HT-29)^33,34^ and sensitive (H-508)^35^ to 5-FU administration as a function of time. Significant differences between SNR values allow us to infer the potential utility of ^19^F CSI to detect resistance of colorectal cancer to 5-FU treatment and potentially guide clinical personalized medicine.

## Results

A total of 23 mice were imaged: 9 mice with the HT-29 tumour (non-responder), 9 mice with the H-508 tumour (responder), and 5 mice with both tumour types. For each animal, the SNR from the tumour, bladder and liver voxels were calculated.

The obtained values were used for plotting the SNR time-dependence curves to detect the presence or absence of 5-FU retention in given tumours. If any organ or tumor was bigger than a single voxel, a mean value of the SNR from all voxels containing the organ or tumor was calculated and used in the subsequent analysis.

**Figure 1:**
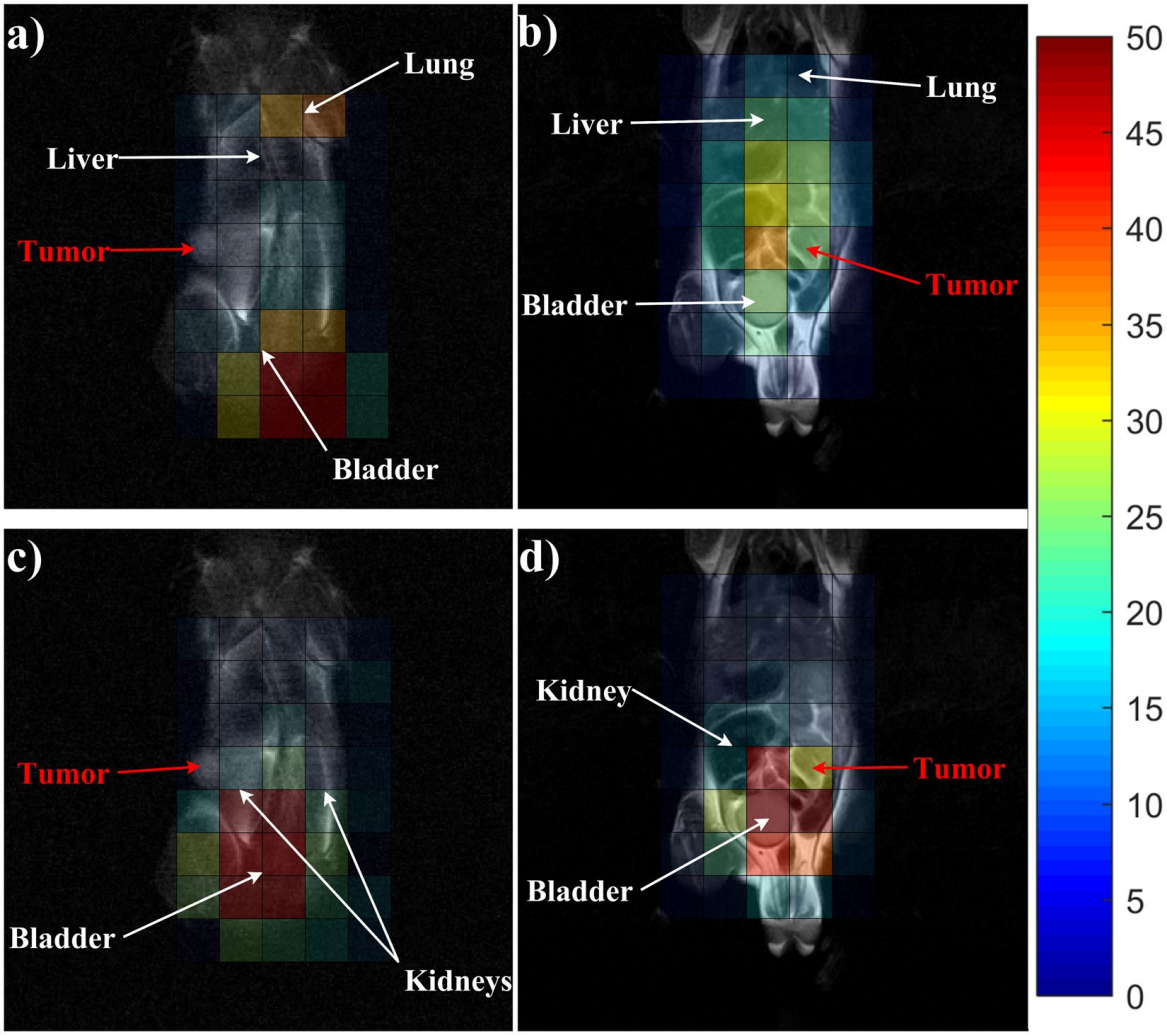
^19^F CSI superimposed onto ^1^H localizer images. (**a**,**c**) represent ^19^F CSI of the HT-29 (non 5-FU responsive) tumour mouse. (**a**,**d**) correspond to CSI of the H-508 (5-FU responsive) tumour mouse. The colour bar represents 5-FU SNR. (**a**,**b**) were acquired at 7.5 minutes after bolus injection, whereas (**c**,**d**) were acquired at 40 minutes after 5-FU injection. At 40 minutes, the signal was concentrated in the H-508 tumor, bladder and kidneys. Notice that the SNR from the H-508 tumour in (**d**) increased by 36% after 40 minutes.

Figure 1 represents the ^19^F CSI images acquired at 7.5(a & b) and 40 minutes (c & d) after bolus injection, superimposed on the ^1^H scans for two representative mice. HT-29 tumour cells were injected into mouse 1 (Fig. 1a,c), whereas mouse 2 (Fig. 1b,d) received H-508 tumour cells. ^19^F SNR values higher than 50 (attributed to bladder uptake) were thresholded to create necessary contrast in tumour voxels between the images acquired at 7.5 and 40 minutes after bolus injection.

The SNR of the HT-29 tumor was 9 at 7.5 minutes after injection (Fig. 1a). The SNR from the H-508 tumour voxel was 22.6 at the same time (Fig. 1b). After 40 minutes, the SNR value from the H-29 tumour decreased slightly and was equal to 7.2 (Fig. 1c), whereas the H-508 tumour SNR increased by 36% and was equal to 30.8 (Fig. 1d).

The liver SNR from the H-508 mouse decreased gradually from 17.8 to 8.3 throughout time. However, the SNR from the liver voxels in mouse 1 was equal to approximately 11 at 7.5 minutes after bolus and 10 at 40 minutes after injection. Bladder SNR was equal to 24.7 and 25.7 for mouse 1 and mouse 2 respectively at 7.5 minutes after injection. At the end of the time period, bladder SNR was equal to 86 for the HT-29 mouse and 58 for the H-508 mouse.

**Figure 2:**
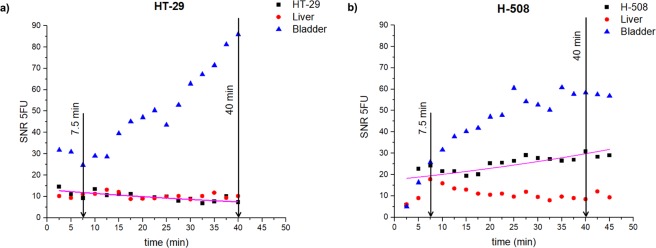
Time course of 5-FU SNR from the tumour, liver, and bladder voxels for representative mouse with a HT-29 tumour (**a**) and mouse with a H-508 tumour. (**b**) The HT-29 (non-responder) SNR steady decreased throughout the time interval (**a**), whereas SNR from the H-508 (tumor responder) voxel increased gradually. (**b**) The pink curve represents the exponential fit (Eq. 1) of the tumour SNR curves.

Figure 2 illustrates the 5-FU SNR time dependences in the tumour, liver, and bladder voxels from representative images of HT–29 (Fig. 2a) and H-508 (Fig. 2b) tumours shown in Fig. 1. The HT-29 tumour SNR decreased steadily throughout time. On the contrary, the SNR from the H-508 tumour voxel grew gradually. The bladder signal steadily increased over time. The liver signal from the HT-29 mouse slightly oscillated around the mean value, which was equal to 10. However, the SNR of the liver voxels of mouse 2 significantly grew from 2.5 to 7.5 minutes after bolus. After 7.5 minutes, the SNR declined gradually.

**Figure 3:**
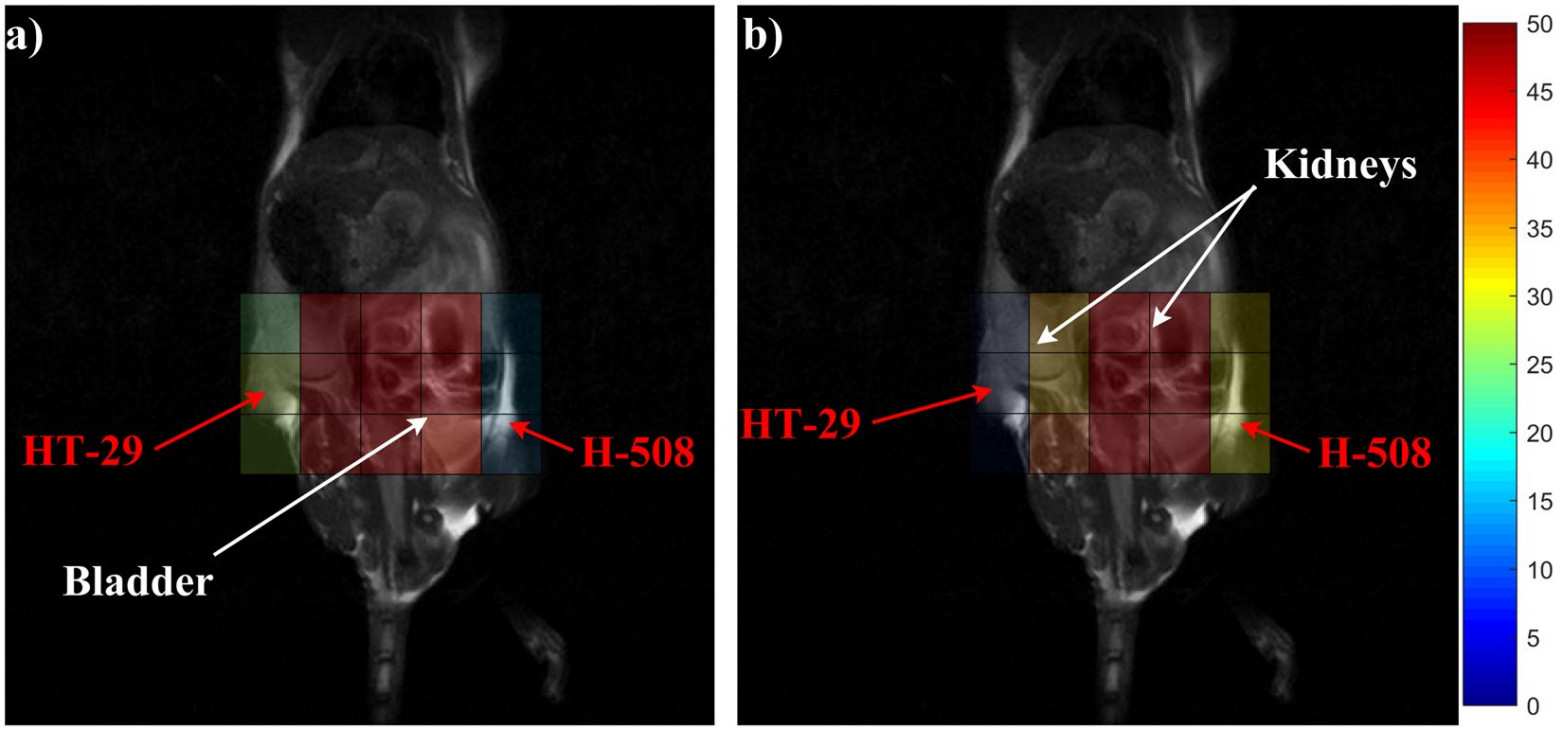
^19^F CSI superimposed onto ^1^H localizer images. Images (**a**,**b**) show a representative mouse with both HT-29 (non 5-FU responsive, injected in the left flank of the animal) and H-508 (5-FU responsive, right flank) tumours at 5 and 70 minutes after 5-FU injection, respectively. The colour bar represents 5-FU SNR. After 70 minutes, high 5-FU signal was acquired from the H-508 tumour, kidneys and bladder. (**b**) Note that the H-508 SNR after 70 minutes was approximately two times higher than at the beginning of study, whereas HT-29 SNR dropped more than 3 times (**b**) comparing to the initial value (**a**).

Figure 3 shows the ^19^F CSIs of mouse 3 superimposed on proton scans. This mouse had both tumour types. The HT-29 tumour was injected into the left flank of the animal whereas the H-508 colorectal adenocarcinoma was injected into the right flank. There are no liver voxels on these CSI images. ^19^F SNR values higher than 50 were thresholded to visually create necessary contrast in tumor voxels between the figures acquired at different times.

Figure 3a,b were acquired at 5 and 70 minutes after bolus injection. The SNRs from the HT-29 and the H-508 voxels at 5 minutes after injection were 26.6 and 12.8 respectively (Fig. 3a). After 70 minutes, the SNR from the HT-29 tumor was 3.4-fold lower than the initial value and equal to 7.9. However, SNR from the H-508 tumour was equal to 31.1 at 70 minutes after bolus. The SNR from the bladder voxels in Fig. 3a was approximately 44, whereas after 70 minutes the SNR was 81.4, which is almost 2 times higher than the signal obtained at 5 minutes after the bolus (Fig. 3b).

**Figure 4:**
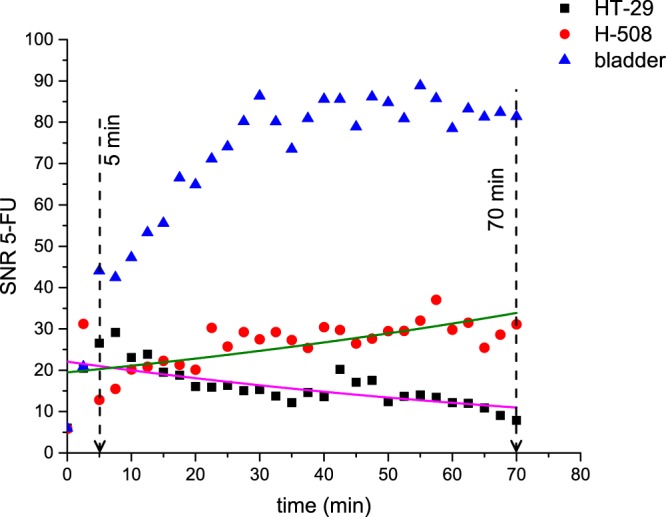
5-FU SNR time curves from the HT-29 (left tumour) (Fig. 3a), the H-508 (right tumour) (Fig. 3b) and the bladder of representative mouse which had both tumor types. The pink and green curves correspond to the exponential fit of the HT-29 and H-508 SNR values respectively. Notice that the HT-29 (non 5-FU responsive tumour) SNR dropped gradually during time period from 10 to 70 minutes after bolus. On the contrary, the H-508 (5-FU responsive tumour) SNR grew steadily throughout the first 22.5 minutes.

Figure 4 shows the time curve evolution of SNR from both tumour types and the bladder. The HT-29 tumour signal increased during the first 10 minutes after 5-FU injection. Nevertheless, SNR decreased steadily throughout time from 10 to 70 minutes after bolus. On the contrary, the signal from the H-508 tumor increased gradually up to 22.5 minutes. The bladder signal grew steadily throughout the first 30 minutes and then leveled. The difference between the HT-29 and the H-508 SNR time curves was statistically significant according to Wilcoxon signed rank test (*p* < 0.01). After 30 minutes, bladder time dependency plateaued at a value of 83 with minor oscillations.

All tumour SNR time curves were fitted using an exponential function with two fitting parameters – amplitude “a” and time constant “b” (Eq. 1). One HT-29 tumour curve was excluded from analysis due to poor goodness of fit. In one of the animals which was injected with both tumour types, the H-508 tumour was not detected. Therefore, just the HT-29 tumour curve was measured. Table 1 represents the values of the time constants “b” obtained for the HT-29 and H-508 singe-tumour mice. The values shown in Table 1 has been used for statistical evaluation of the obtained results. If the mean value of the observed time constants is positive and significantly different from a zero value, the tumour can successfully retain 5FU.

**Table 1:** The time constants “b” (min^−1^) obtained from the exponential fit of HT-29 and H-508 SNR time curves of single-tumour mice.

	HT-29	H-508
1	−0.014 ± 0.003	0.013 ± 0.006
2	−0.006 ± 0.005	0.028 ± 0.007
3	0.007 ± 0.004	0.013 ± 0.003
4	−0.011 ± 0.0004	0.044 ± 0.008
5	0.0003 ± 0.001	0.005 ± 0.006
6	0.009 ± 0.002	0.034 ± 0.008
7	−0.007 ± 0.003	0.011 ± 0.009
8	−0.006 ± 0.003	−0.002 ± 0.001
9	−0.012 ± 0.002	0.017 ± 0.003
Mean ± SD	−0.004 ± 0.008	0.018 ± 0.015

**Figure 5:**
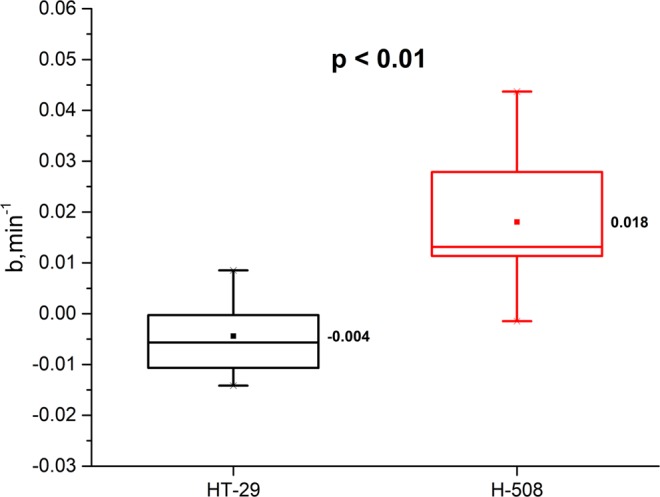
Box chart of the time constant for the HT-29 (non 5-FU responder) and the H-508 (5-FU trapper) tumours of single-tumour mice group. Mean value of the time constant for the HT-29 tumour was equal to −4 × 10^−3^ min^−1^, however for the H-508 tumour mean time constant was equal to 18 × 10^−3^min^−1^.

The mean value + /− one standard deviation of the time constants for the HT-29 tumour in single-tumor mice was equal to -0.004 ± 0.008 min^−1^. On the contrary, the mean time constant *“b”* for the H-508 tumour was equal to 0.018 ± 0.015 min^−1^. The results are significantly different according to two-sample unpaired t-test (*p* < 0.01). Furthermore, according to one-sample t-test, the mean value for the H-508 tumour is significantly greater than 0 (*p* < 0.01). Figure 5 shows a box chart analysis of time constants from both tumour types with a significant difference of *p* < 0.01 between the tumour types. Based on this statistical analysis, we can conclude that 5-FU uptake of studied tumors was significantly different.

The mean values of the time constant “b” for the H-508 and HT-29 tumors of dual-tumour cohort were equal to 0.006 ± 0.009 min^−1^ and −0.001 ± 0.008 min^−1^ respectively. The difference in mean values was not statistically significant (p > 0.01). However, the Wilcoxon test showed that SNR curves of two different tumours from the dual-tumour animals were significantly different (p < 0.01).

## Discussion

The results of this study illustrate the feasibility of detecting a 5-FU retention in different types of colorectal cancers using ^19^F CSI imaging. Time curves of the 5-FU signal acquired following bolus injection can reveal the difference in uptake of 5-FU in different tumour types. Time curves of the 5-FU signal acquired right after a bolus injection of the chemotherapy drug had different dynamics for two different types of human colon adenocarcinoma. Therefore, detection of tumour resistance to chemotherapy based on a 5-FU retention is possible approximately one hour after bolus injection.

5-FU SNR changes with time were found to be significantly different (*p* < 0.01) for the HT-29 and H-508 tumour types. Increasing 5-FU SNR throughout time is characteristic of the H-508 tumour, whereas the HT-29 tumour type SNR shows a tendency to decrease or to remain constant. This result was obtained for the single-tumor mice and it was statistically significant (p < 0.01). A significant difference between time constant “b” for the dual-tumour animals was not observed (p > 0.01). It could be due to a small number of samples (n = 5). These results are consistent with the literature which shows that the HT-29 tumor demonstrates resistivity to 5-FU therapy while the H-508 tumor can be treated using 5-FU^33–35^.

We did not observe 5-FU “trapping phenomenon” as described by Presant during the studies timeline from both tumour types. For the H-508 type, the signal did not reach steady-state, so we were not able to estimate the half-life time for the 5-FU in the tumor. However, the retention of 5-FU in H-508 tumour was observed over the studied time course. The HT-29 tumour is not expected to retain 5-FU due to the observation of the negative b time constant indicating a decline in 5-FU within the tumour.

5-FU SNR within the bladder increased steadily from injection time to 70 minutes after treatment. Nearly all of the bladder 5-FU SNR was observed to be stronger than the tumour and liver SNRs. This finding could be due to catabolism of 5-FU in the liver. The limiting factor of 5-FU metabolism in the liver could be attributable to the activity of the enzyme dihydrouracil dehydrogenase^5,24^. Indeed, Fig. 2 illustrates the decay of the liver SNR with time. This time course corroborates the results, obtained in the rat model^32^. The control animals which received no cell injection had the same SNR dynamics for the liver and bladder.

Unfortunately, we were not able to observe the signals of 5-FU metabolites such as Fbal and Fnuc. 5-FU catabolizes into Fnuc in the tumour^5,27,32^, whereas Fbal can be found in the liver, tumor, and kidneys^5,23,27,32^. According to Otake (2011), Fnuc signal was observed 1 hour after bolus and Fbal signal was detected 10 minutes after injection^32^. In that study, the authors used a 7.0T animal MRI system. The absence of a ^19^F signal from the fluorinated metabolites could be due to the small concentration of these metabolites in the organs and tumours. Our study time interval was likely too short to allow for a high concentration of catabolites to be produced. Future imaging of the metabolites Fbal and Fnuc requires further parameter optimization due to the low SNR of these metabolites even 40 minutes after bolus. Additionally, to improve the sensitivity of the RF coil, a phased array coil could be used for better coupling with ^19^F nuclei in the tumor and organs.

Overall, our studies demonstrate that ^19^F CSI imaging can be used to detect retention of 5-Fluorouracil in a murine model of colorectal cancer. This technique may extend prediction of patient tumour resistivity to 5-FU chemotherapy based on the pharmacokinetics of 5-FU at an early treatment stage, yielding an improvement in personalized cancer therapy. Implementing innovative imaging strategies that identify patients who respond to 5-FU would increase the efficacy of the treatment by allowing targeted matching of patients and chemotherapy agents in the neoadjuvant setting. This MRI-based technique could be readily implemented clinically as a means of non-invasively monitoring early response to 5-FU chemotherapy. Identifying resistant tumours early in cancer treatment will enable patients unlikely to respond to 5-FU therapy to rapidly alter treatment plans. The use of ^19^F CSI to triage patients into different chemotherapy regimens could be a significant innovation and alter routine clinical practice.

## Materials and Methods

### Cell lines and culture conditions

All human colon adenocarcinoma cell lines, media, sera, and culture reagents were obtained from ATCC (Burlington, ON, Canada), Life Technologies (Burlington, Ontario, Canada), Becton Dickinson (St. Laurent, Quebec, Canada) or Sigma (St. Louis, MO). HT-29 cells were grown in McCoy’s 5 A medium and NCI-H508 were grown in RPMI-1640 medium, with both cell lines supplemented with 10% FBS, 100U/ml Penicillin, 100 µg/ml streptomycin, and 2 mM glutamine. Cells were grown to 50–75% confluency in T-75 flasks prior to injection into animals.

### *In Vivo* cell implantation and tumour growth in immunodeficient mice

This study was approved by Lakehead University Animal Care Committee, and all procedures were done in compliance with the regulations of the Canadian Counsel on Animal Care (CCAC). HT-29 and NCI-H508 cells were grown in T-75 flasks between 60–80% confluency. Cells were trypsinized with 0.25% (w/v) Trypsin −0.53 mM EDTA solution, resuspended in appropriate medium, and counted. 1 × 10^6^ human colon adenocarcinoma cells were mixed with a 1:1 ratio cold Matrigel (Corning Matrigel, Fisher Scientific) for each 100 µl bolus injection into 36–40 day-old male (Nu/Nu) nude mice.

Briefly, nude mice were aestheticized using 3% isoflurane and injected with 100ul of a 1:1 cold Matrigel solution of either 1 × 10^6^ HT-29 or NCI-H508 cells using a BD Eclipse 27 G × 1/2 needle. Mice were injected under the skin in the left (HT-29) or right flank (NCI-H508), weighed, labelled by ear piercing, and immediately returned to cages. Mock mice were injected similarly with 100 µl of a 1:1 ratio of appropriate cold medium and Matrigel while control mice received no injection. The H-508 tumour grew more slowly than HT-29. Therefore, the dual-tumour mice were injected with HT-29 tumor 5 days post HT-508 cells injection. A total of 23 mice were injected with either HT-29 cells (9 mice), H – 508 cells (9 mice) or both adenocarcinoma types (5 mice). All animal imaging was performed 12–16 days post injection or when a caliper measurement of the volume of the tumours did not exceed either 450 ± 75 mm^3^ for animals with single tumours or 600 ± 75 mm^3^ in total for the dual-tumour mice. The mouse body weight was between 30–37 g.

### MRI acquisition

Prior to the MRI scanning procedure, all animals were anesthetized using 5% isoflurane oxygen mixture and anesthesia was maintained at 2% with oxygen during all subsequent experiments. Mice were catheterized using a MTV-01 tail-vein catheter (SAI Instruments). All animals received a slow bolus injection of 300 µl 5-FU (50 mg/ml) over the course of 2 minutes inside the magnet bore. During each MRI acquisition, animals were kept at 37 C° with a temperature-controlled water-filled blanket (T/Pump, Gaymar).

MRI was performed using a clinical Philips 3.0 T Achieva whole-body scanner, equipped with a custom-built dual-tuned ^1^H/^19^F quadrature birdcage coil. Proton localization was performed using a multi-slice T_1_-weighted Turbo Spin Echo (TSE) pulse sequence with a Field of View (FOV) of 75 × 75 mm^2^, TR/TE = 2000/55.19 ms, slice thickness of 2 mm and Number of Signal Averages (NSA) = 3. Acquisition matrix size was equal to 256 × 256 which corresponds to the in-plane resolution of 0.29 mm. The total number of slices was equal to 16 for each mouse. Proton scans were used to determine the location of the tumour, bladder and liver.

After the bolus injection, ^19^F CSI images were acquired for up to 70 minutes with a time step of 2 minutes and 30 seconds. Mice that had one tumour type were studied using CSI with 8 × 5 resolution, FOV of 20 × 50 mm^2^ and NSA = 3, whereas mice with both tumour types were studied using 3 × 5 matrix, FOV equal to 31 × 18.6 mm^2^ and NSA = 9. All CSI images were acquired using spectral bandwidth of 32 kHz (266 ppm at 3.0 T) and TR/TE = 5000/4.27 ms. The data sampling number was 1024, yielding a spectral resolution of 0.26 ppm.

Images were analyzed using a custom imaging processing program written in MATLAB R2016b (The Mathworks, Inc, Natick, MA).

### Statistical analysis

All SNR time curves were fitted by the exponential function

1 $$SNR=a{{\rm{e}}}^{bt}$$

where amplitude *a* and time constant *b* were fitting parameters. The time constant determines signal dynamics. If the time constant is positive, the signal will grow with time, conversely when it is negative, signal will decrease. Thus, time constant can be used as an indicator of 5-FU kinetics. A two-sample t-test was applied for *b* time constant to analyze the statistical significance of the fitted results for the single tumour animals. A one-sample t-test was applied to the *b* time constant of each group of single tumour animals to evaluate if the mean *b* value of each group was significantly different from 0. A Wilcoxon signed rank test has been used to evaluate the difference between SNR time curves obtained from different tumors in dual-tumor animals. OriginPro 2016 was used to conduct statistical analysis (OriginLab Corp., Northampton, MA).

## References

[1] World Healthcare Organisation. Cancer. 2018. 1–7 Available at, http://www.who.int/mediacentre/factsheets/fs297/en/ (2018).

[2] Colorectal cancer statistics - Canadian Cancer Society. Available at, http://www.cancer.ca/en/cancer-information/cancer-type/colorectal/statistics/?region=on.

[3] Canadian Cancer Statistics 2017 Special topic: Pancreatic cancer. Available at, http://www.cancer.ca/~/media/cancer.ca/CW/cancer information/cancer101/Canadian cancer statistics/Canadian-Cancer-Statistics-2017-EN.pdf?la=en.

[4] Brenner DR (2017). Increasing colorectal cancer incidence trends among younger adults in Canada. Prev. Med. (Baltim).

[5] Longley DB, Harkin DP, Johnston PG (2003). 5-Fluorouracil: mechanisms of action and clinical strategies. Nat. Rev. Cancer.

[6] Midgley R, Kerr D (1999). Colorectal Cancer. Lancet.

[7] McIntyre DJO (2011). Can localised 19F magnetic resonance spectroscopy pharmacokinetics of 5FU in colorectal metastases predict clinical response?. Cancer Chemother. Pharmacol..

[8] Folprecht G (2004). Efficacy of 5-fluorouracil-based chemotherapy in elderly patients with metastatic colorectal cancer: A pooled analysis of clinical trials. Ann. Oncol.

[9] Launay M (2016). Beating the odds: Efficacy and toxicity of dihydropyrimidine dehydrogenase-driven adaptive dosing of 5-FU in patients with digestive cancer. Br. J. Clin. Pharmacol..

[10] Arbuck SG (1989). Overview of clinical trials using 5-fluorouracil and leucovorin for the treatment of colorectal cancer. Cancer.

[11] Odin E, Sondén A, Gustavsson B, Goran C, Yvonne W (2015). Expression of Folate Pathway Genes in Stage III Colorectal Cancer Correlates with Recurrence Status Following Adjuvant Bolus 5-FU-Based Chemotherapy. Mol. Med..

[12] Souglakos J (2006). FOLFOXIRI (folinic acid, 5-fluorouracil, oxaliplatin and irinotecan) vs FOLFIRI (folinic acid, 5-fluorouracil and irinotecan) as first-line treatment in metastatic colorectal cancer (MCC): A multicentre randomised phase III trial from the Hellenic Oncolog. Br. J. Cancer.

[13] Saltz LB (2002). Irinotecan plus Fluorouracil and Leucovorin for Metastatic Colorectal Cancer. N. Engl. J. Med..

[14] Douillard J (2000). Irinotecan combined with fluorouracil compared with fluorouracil alone as first-line treatment for metastatic colorectal cancer: a multicentre randomised trial. Lancet.

[15] Presant CA (1994). Association of intratumoral pharmacokinetics of fluorouracil with clinical response. Lancet.

[16] Jung SH (2012). Predicting response to neoadjuvant chemoradiation therapy in locally advanced rectal cancer: Diffusion-weighted 3 tesla MR imaging. J. Magn. Reson. Imaging.

[17] Marugami N (2009). Early detection of therapeutic response to hepatic arterial infusion chemotherapy of liver metastases from colorectal cancer using diffusion-weighted MR imaging. Cardiovasc. Intervent. Radiol..

[18] Lavdas I (2018). Histogram analysis of apparent diffusion coefficient from whole-body diffusion-weighted MRI to predict early response to chemotherapy in patients with metastatic colorectal cancer: preliminary results. Clin. Radiol..

[19] Soujanya Chilla G, Heng Tan C, Xu C, Loo Poh C (2015). Diffusion weighted magnetic resonance imaging and its recent trend-a survey. Quant Imaging Med Surg.

[20] Baliyan V, Das CJ, Sharma R, Gupta AK (2016). Diffusion weighted imaging: Technique and applications. World J. Radiol..

[21] Wolf W, Waluch V, Presant CA (1998). Non-invasive 19F-NMRS of 5-fluorouracil in pharmacokinetics and pharmacodynamic studies. NMR Biomed..

[22] Lovis, J. A. *et al*. Monitoring *in-vivo* Absorption of 5-Fluorouracil by 19 F MRI: A Preliminary Study for Clinical Pharmacokinetics Using a Clinical MRI System. *In Proc. ISMRM 2592*.

[23] Gade TPF (2004). *In vivo* 5-fluorouracil and fluoronucleotide T1 relaxation time measurements using the variable nutation angle method. Magn. Reson. Med..

[24] Brix G (1995). Mapping the Biodistribution and Catabolism of 5-Fluorouracil in Tumor-Bearing Rats by Chemical-Shift Selective 19F MR Imaging. Magn. Reson. Med..

[25] Brix G, Bellemann ME, Haberkorn U, Gerlach L, Lorenz WJ (1996). Assessment of the biodistribution and metabolism of 5-fluorouracil as monitored by 18F PET and 19F MRI: A comparative animal study. Nucl. Med. Biol..

[26] Doi, Y. *et al*. 19F Chemical Shift Imaging of F-nuc Formed from 5-FU in Mouse Tumor by Fast Spin Echo. In *Spatially Resolved Magnetic Resonance Methods, Materials, Medicine, Biology,Rheology, Geology, Ecology, Hardware* (eds Blümler, P., Blumich, B., Botto, R. & Fukushima, E.) 413–419 (Wiley-VCH Verlag GmbH, 1988).

[27] Doi Y, Shimmura T, Kuribayashi H, Tanaka Y, Kanazawa Y (2009). Quantitative 19F imaging of nmol-level F-nucleotides/-sides from 5-FU with T2 mapping in mice at 9.4T. Magn. Reson. Med..

[28] Kuribayashi H, Doi Y, Kanazawa Y (2001). Application of (19)F chemical shift imaging in studies of mice with orally administered 5-fluorouracil. Magn. Reson. Med..

[29] Li C-W (1996). Quantitation of 5-Fluorouracil Catabolism in Human Liver *in Vivo* by Three-Dimensional Localized 19F Magnetic Resonance Spectroscopy 1. Clin. Cancer Res..

[30] Klomp Dennis W.J., van Laarhoven Hanneke W.M., Kentgens Arno P.M., Heerschap Arend (2003). Optimization of localized19F magnetic resonance spectroscopy for the detection of fluorinated drugs in the human liver. Magnetic Resonance in Medicine.

[31] van Laarhoven H. W. M., Klomp D. W. J., Rijpkema M., Kamm Y. L. M., Wagener D. J. Th., Barentsz J. O., Punt C. J. A., Heerschap A. (2007). Prediction of chemotherapeutic response of colorectal liver metastases with dynamic gadolinium-DTPA-enhanced MRI and localized19F MRS pharmacokinetic studies of 5-fluorouracil. NMR in Biomedicine.

[32] Otake, Y., Hirata, K., Soutome, Y. & Bito, Y. In-vivo 19 F Imaging of 5-Fluorouracil and its Metabolites in Rat by Two-Element Phased-Array Coil. In *Proc. Intl. Soc. Mag. Reson. Med. 19* (2011).

[33] Lesuffleur T (1998). Resistance to high concentrations of methotrexate and 5-fluorouracil of differentiated HT-29 colon-cancer cells is restricted to cells of enterocytic phenotype. Int. J. Cancer.

[34] Denise C (2015). 5-Fluorouracil resistant colon cancer cells are addicted to OXPHOS to survive and enhance stem-like traits. Oncotarget.

[35] Park J-G (1987). Characteristics of cell lines established from human colorectal carcinoma. Cancer Res..

